# Wax Candle in Bladder of Female

**Published:** 1873-11

**Authors:** 


A Wax Candle in the Bladder of a Female.—This interesting case was observed a short time ago at the Hotel Dieu, of Paris. The patient on admission complained of intense pain in the abdomen. The urethra, abnormally dilated, easily allowed of the introduction of a finger into the bladder, where there was felt a hard, voluminous body, obviously the cause of the intense pain. The woman then mentioned that, on account of a great difficulty in making water, she had passed a candle through the urethra, and had let it fall accidentally in the bladder. Through the extraordinary dilatation of the urethra, pincers were easily passed through the canal, and after some efforts the candle was caught by the wick and drawn out through the urethra. The end of the candle (which had been rounded with a knife) was seen to be covered with calcareous matter, which had gathered there during the five weeks which the candle had stayed in the bladder. A few days after the extraction the woman left the hospital, perfectly recovered.

